# Statement of Retraction

**DOI:** 10.1080/21655979.2026.2614863

**Published:** 2026-01-19

We, the Editor-in-Chief and Publisher of *Bioengineered (the “**Journal**”)*, have retracted the following articles which were published in the Special Issue titled “***Algal Bioprocess Engineering****”*, guest edited by Pau Loke Show, Kit Wayne Chew & Jo-Shu Chang, *Bioengineered, 11(S12), originally published in 10(01) and 11(01)*.

- Show, P. L., Chew, K. W., & Chang, J. S. (2020). Special issue on algae bioprocess engineering. *Bioengineered, 11*(SI2), 188. https://doi.org/10.1080/21655979.2020.1729546
- Koyande, A. K., Show, P. L., Guo, R., Tang, B., Ogino, C., & Chang, J. S. (2019). Bio-processing of algal bio-refinery: a review on current advances and future perspectives. *Bioengineered, 11*(SI2), 574–592. https://doi.org/10.1080/21655979.2019.1679697
- Ghanbariasad, A., Taghizadeh, S. M., Show, P. L., Nomanbhay, S., Berenjian, A., Ghasemi, Y., & Ebrahiminezhad, A. (2019). Controlled synthesis of iron oxyhydroxide (FeOOH) nanoparticles using secretory compounds from Chlorella vulgaris microalgae. Bioengineered, 11(SI2), 390–396. https://doi.org/10.1080/21655979.2019.1661692
- Cheah, W. Y., Show, P. L., Yap, Y. J., Mohd Zaid, H. F., Lam, M. K., Lim, J. W., … Tao, Y. (2019). Enhancing microalga Chlorella sorokiniana CY-1 biomass and lipid production in palm oil mill effluent (POME) using novel-designed photobioreactor. Bioengineered, 11(SI2), 61–69. https://doi.org/10.1080/21655979.2019.1704536
- Tan, J. S., Lee, S. Y., Chew, K. W., Lam, M. K., Lim, J. W., Ho, S. H., & Show, P. L. (2020). A review on microalgae cultivation and harvesting, and their biomass extraction processing using ionic liquids. Bioengineered, 11(SI2), 116–129. https://doi.org/10.1080/21655979.2020.1711626
- Taghizadeh, S. M., Berenjian, A., Chew, K. W., Show, P. L., Mohd Zaid, H. F., Ramezani, H., … Ebrahiminezhad, A. (2020). Impact of magnetic immobilization on the cell physiology of green unicellular algae *Chlorella vulgaris. Bioengineered, 11*(SI2), 141–153. https://doi.org/10.1080/21655979.2020.1718477

Following publication, the Publisher identified concerns regarding the editorial handling of the Special Issue and the peer review process.

An investigation by the Taylor & Francis Publishing Ethics & Integrity team in full cooperation with the Editor-in-Chief concluded that the articles included in this Special Issue were not peer-reviewed appropriately, in line with the Journal’s peer review standards and policy.

As the stringency of the peer review process is core to the integrity of the publication process, the Editor-in-Chief and Publisher have decided to retract all the articles within the above-named Special Issue. The Editor-in-Chief and the Publisher have not confirmed if all the authors were aware of the concerns with the peer review process.

The Journal is committed to correcting the scientific record and will fully cooperate with any institutional investigations into this matter. The authors have been informed of this decision.

We have been informed in our decision-making by our editorial policies and the COPE guidelines.

The retracted articles will remain online to maintain the scholarly record, but they will be digitally watermarked on each page as ‘Retracted’.

